# Supplementary material for: Recovery From Severe Mental Health Problems: A Systematic Review of Service User and Informal Caregiver Perspectives
Source: Front Psychiatry. 2021 Sep 1;12:712026. doi: 10.3389/fpsyt.2021.712026 (PMC8440827; doi:10.3389/fpsyt.2021.712026)
Supplement: Supplementary file 3 [file Data_Sheet_3.docx]

Supplementary File 3. Risk of bias assessment

Quality Assessment Checklist

Epistemological soundness

Are the research question/objectives clear?

Is a qualitative approach appropriate/justified to answer the research question?

Characteristics of the study

Are the sources of data /population adequately described?

Is sampling strategy described?

Is there coherence between objectives – results - discussion?

Are research questions and objectives clear and focused?

Has the relationship between researcher and participants been considered?

References

Hong QN, Pluye P, Fàbregues S, Bartlett G, Boardman F, Cargo M, *et al.* Mixed Methods Appraisal Tool (MMAT) Version 2018 User guide. , 2018 (http://mixedmethodsappraisaltoolpublic.pbworks.com/).

Critical Appraisal Skills Programme (CASP) Checklist. (https://casp-uk.net/wp- content/uploads/2018/01/CASP-Systematic-Review-Checklist_2018.pdf).

Bromley H, Dockery G, Fenton C, Nhlema B, Smith H, Tolhurst R, *et al.* LSTM. Criteria for Evaluating Qualitative Studies. , 2002 (https://www.depts.ttu.edu/education/our- people/Faculty/additional_pages/duemer/epsy_5382_class_materials/Evaluating-Qualitative- Studies.pdf).

Dixon-Woods M, Shaw RL, Agarwal S, Smith JA. The problem of appraising qualitative research. *Qual Saf Health Care* 2004; **13**: 223–5.
